# Structural damage burden and hypertrophic olivary degeneration in pediatric postoperative cerebellar mutism syndrome

**DOI:** 10.1007/s10143-022-01791-7

**Published:** 2022-04-20

**Authors:** Thomas Beez, Christopher Munoz-Bendix, Hendrik-Jan Mijderwijk, Marc Remke, Daniel Hänggi

**Affiliations:** 1 Department of Neurosurgery, Medical Faculty, Heinrich-Heine-University, Moorenstrasse 5, 40225 Düsseldorf, Germany; 2 Department of Pediatric Oncology, Hematology and Clinical Immunology, Medical Faculty, Heinrich-Heine-University, Düsseldorf, Germany

**Keywords:** Cerebellar mutism syndrome, Posterior fossa tumor, Children, Hypertrophic olivary degeneration, Dentate nucleus, Superior cerebellar peduncle, Personalized medicine

## Abstract

Cerebellar mutism syndrome (CMS) occurs in one out of four children after posterior fossa tumor surgery, with open questions regarding risk factors, pathophysiology, and prevention strategies. Because of similarities between several cerebellar syndromes, a common pathophysiology with damage to the dentato-thalamo-cortical and dentato-rubro-olivary pathways has been proposed. Hypertrophic olivary degeneration (HOD) is an imaging correlate of cerebellar injury observed for instance in stroke patients. Aim of this study was to investigate whether the occurrence and severity of CMS correlates with the extent of damage to the relevant anatomical structures and whether HOD is a time-dependent postoperative neuroimaging correlate of CMS. We performed a retrospective single center study of CMS patients compared with matched non-CMS controls. CMS occurred in 10 children (13% of the overall cohort) with a median age of 8 years. Dentate nucleus (DN) injury significantly correlated with CMS, and superior cerebellar peduncle (SCP) injury was associated by tendency. HOD was observed as a dynamic neuroimaging phenomenon in the postoperative course and its presence significantly correlated with CMS and DN injury. Children who later developed HOD had an earlier onset and tended to have longer persistence of CMS. These findings can guide surgical measures to protect the DN and SCP during posterior fossa tumor resections and to avoid a high damage burden (i.e., bilateral damage). Development of intraoperative neuromonitoring of the cerebellar efferent pathways as well as improved preoperative risk stratification could help to establish a patient-specific strategy with optimal balance between degree of resection and functional integrity.

## Introduction

Cerebellar mutism syndrome (CMS) occurs in one out of four children after posterior fossa tumor surgery, with considerable variation across published studies [6, 26]. It is defined as a postoperative syndrome clinically characterized by delayed onset of mutism and emotional lability, which can occur in combination with muscular hypotonia, dysphagia, and cerebellar cognitive affective syndrome and cerebellar motor syndrome, and is transient, although residual sequelae can persist [14]. Additionally, some authors differentiate between paucity and absence of speech as well as symptom severity and temporal course [13, 15, 16, 27].

The complex neuronal circuitry of the cerebellum was initially investigated with microscopic and electrophysiological techniques [9, 33]. Modern neuroimaging studies using structural (e.g., diffusion tensor imaging, DTI), functional (e.g., functional magnetic resonance imaging, fMRI), and metabolic/perfusion methods (e.g., single-photon emission computerized tomography, SPECT, and arterial spin labelling perfusion MRI, ASL) provided insight into the cerebellum’s connectivity and involvement in a broad spectrum of brain functions [1, 4, 5, 32, 36]. These include motor, cognition, language, and emotion [35]. The functional understanding derived from these studies is reflected in pathological conditions: depending on location in the posterior versus anterior cerebellum, predominantly motor versus cognitive deficits occur after cerebellar ischemic stroke [34]. Imaging correlates of disruption of cerebellar pathways after ischemic and hemorrhagic stroke have been observed, with hypertrophic olivary degeneration (HOD) being well characterized with its specific time course after cerebellar insults [12, 41]. Recognizing similarities between several cerebellar syndromes, including CMS, a common pathophysiology, has been proposed [31]. The contemporary concept of CMS pathophysiology is damage to the dentato-thalamo-cortical and dentato-rubro-olivary pathways [6, 26]. While there is evidence of preoperative functional deficits in affected children, the full clinical picture of CMS only develops after surgery and it is thus a postoperative morbidity or complication [28].

Based on the aforementioned assumptions, we hypothesized that (1) similar to other types of postoperative morbidity, the occurrence and severity of CMS correlates with the extent of damage to the relevant anatomical structures (i.e., structural damage burden), and that (2) similar to other types of cerebellar insults, such as stroke, HOD is a time-dependent postoperative neuroimaging correlate of CMS.

## Materials and methods

This retrospective study was approved by the local ethical review board (study no. 2019–417-RetroDEuA). CMS cases were identified in the institutional pediatric (defined as 16 years of age or younger at time of diagnosis) brain tumor database between 2010 and 2017. To be eligible for inclusion in this study, preoperative and follow-up MRI scans within 72 h after surgery and after that at least every 3 to 6 months for at least 2 years had to be available for analysis. Demographic, clinical, and tumor-associated variables were extracted from the electronic patient database. CMS was graded according to the scale devised by Robertson et al., taking into account time of onset (day 1, days 1–2, days 2–4, or > day 4) as well as severity and duration of mutism, ataxia, muscle hypotonia, and irritability (each graded into mild/ < 1 week, moderate/1–4 weeks, or severe/ > 4 weeks) [27]. The risk for developing CMS based on preoperative MRI was calculated with the Rotterdam Risk Score [8].

Early postoperative MRI scans performed within 72 h after surgery were analyzed concerning T2 signal abnormality in the vermis, dentate nucleus, inferior, middle and superior cerebellar peduncles, inferior olivary nucleus, floor of the IV ventricle, and the midbrain. Serial follow-up MRI scans were analyzed for T2 signal abnormality with regard to volume and signal intensity of the inferior olivary nucleus. These analyses were performed on axial T2-weighted sequences obtained on a 1.5 or 3.0 Tesla MRI scanner (Siemens Healthcare, Erlangen, Germany). HOD was radiologically defined according to methodology established in three published studies [11, 21, 41]. CMS cases were compared with non-CMS controls that were matched for age, gender, and histology.

Data was processed and analyzed using GraphPad Prism for Windows (GraphPad Software, San Diego, USA). Descriptive statistics are provided, with means and medians as indicated. For analyzing the association between groups and outcomes, Fisher’s exact test was used to calculate two-tailed *P* values. A *P* value less than 0.05 was determined a priori to be statistically significant.

## Results

### Baseline characteristics of the study groups

**Fig. 1:**
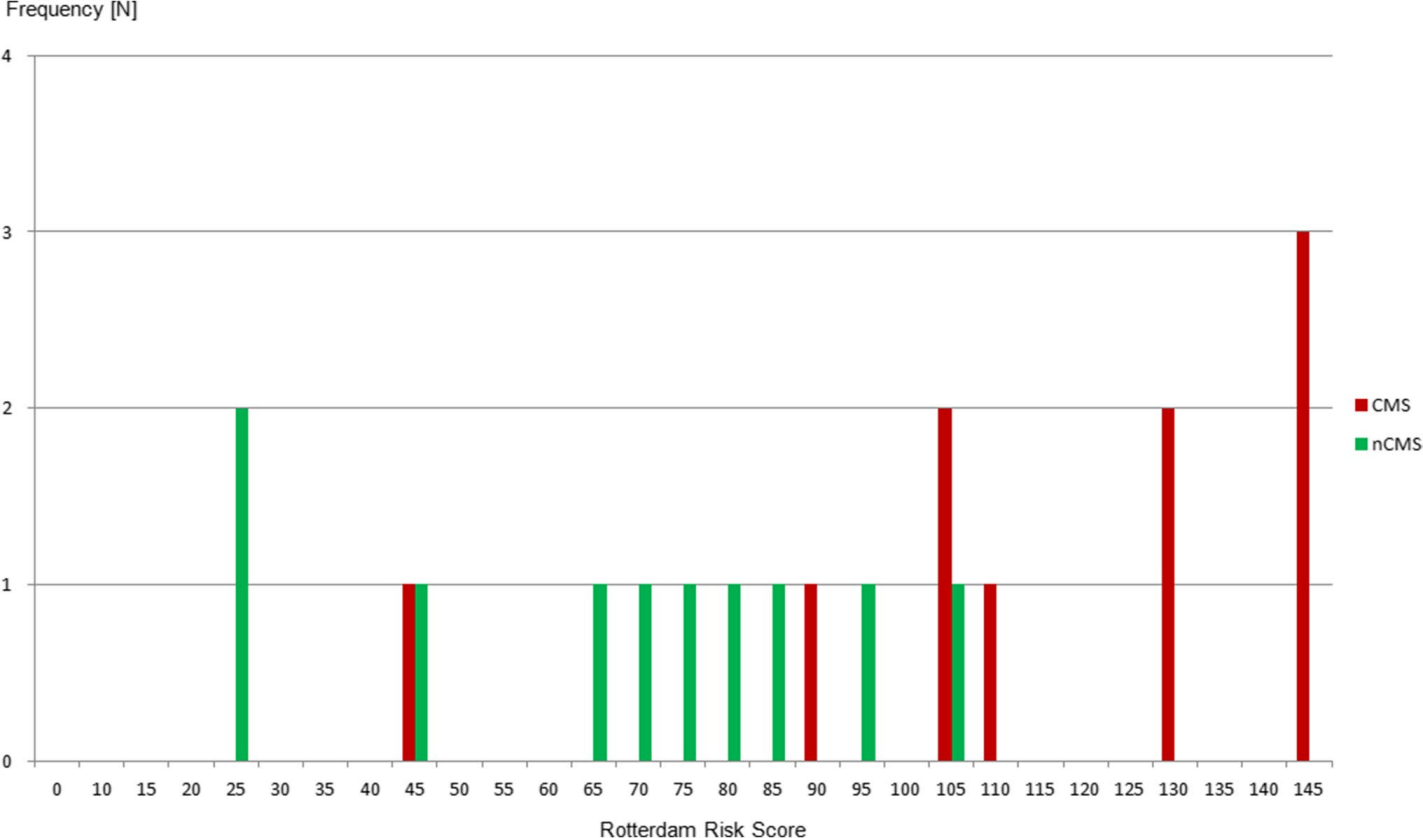
Rotterdam Risk Scores for CMS (red bars) and non-CMS (nCMS, green bars) patients

In an overall cohort of 78 eligible pediatric posterior fossa tumors treated with microsurgical resections using a telovelar approach and intraoperative neuromonitoring during the study period, CMS occurred in 10 children (13% of the overall cohort), comprising 6 boys and 4 girls with a median age of 8 years (range 4 to 14 years). The median age in the non-CMS group was 9.5 years (range 4 to 13 years). Demographic, clinical, and tumor-associated variables of the CMS and non-CMS groups are summarized in Table 1, reflecting balanced groups with regard to baseline parameters. The median tumor volume was 40 ml (range 15 to 114 ml) in the CMS group and 36 ml (range 10 to 76 ml) in the non-CMS group. The median Evans index was 0.37 (range 0.27 to 0.46) and 0.35 (range 0.25 to 0.41), respectively. The median Rotterdam Risk Score was 120 (range 45 to 145) in children who later developed CMS, compared with 72.5 (range 25 to 105) in those who did not (*P* = 0.0019) (Fig. 1). Gross total resections were more common in the CMS (50%) than in the non-CMS group (20%). Histology was medulloblastoma WHO grade IV (*N* = 5), pilocytic astrocytoma WHO grade I (*N* = 3), atypical ependymoma WHO grade II (*N* = 1), and anaplastic ependymoma WHO grade III (*N* = 1), matched in both groups. Data on molecular subgroups was not available for the majority of patients, as they were treated before implementation of the molecular subgroup consensus classifications into our neuropathological routine [22, 38]. Regarding medulloblastoma, all tumors in the CMS group were ß-catenin nucleonegative. In the non-CMS group, medulloblastomas were ß-catenin nucleonegative (*N* = 2), ß-catenin nucleopositive (*N* = 1), and without ß-catenin analysis (*N* = 2).

**Table 1:** Demographic, clinical, and tumor-associated variables of the CMS and non-CMS (nCMS) groups. *PA*, pilocytic astrocytoma; *MB*, medulloblastoma; *EP*, ependymoma; *STR*, subtotal resection; *NTR*, near total resection; *GTR*, gross total resection

CMS group							Non-CMS group						
Patient no	Age [y]	Histology	Tumor volume [ml]	Hydro-cephalus [Evans index]	Rotterdam Risk Score	Degree of resection	Patient no	Age [y]	Histology	Tumor volume [ml]	Hydro-cephalus [Evans index]	Rotterdam Risk Score	Degree of resection
CMS_1	5	PA	30	N [0.27]	105	NTR	nCMS_1	4	PA	31	Y [0.38]	95	GTR
CMS_2	7	MB	15	Y [0.33]	45	STR	nCMS_2	5	MB	10	Y [0.31]	45	NTR
CMS_3	10	PA	42	Y [0.30]	105	NTR	nCMS_3	10	PA	45	N [0.28]	65	STR
CMS_4	10	PA	55	Y [0.39]	90	GTR	nCMS_4	10	PA	76	Y [0.36]	75	NTR
CMS_5	4	EP III	44	Y [0.40]	130	GTR	nCMS_5	3	EP III	40	Y [0.34]	70	NTR
CMS_6	7	MB	40	Y [0.34]	145	GTR	nCMS_6	10	MB	20	N [0.25]	80	STR
CMS_7	8	MB	34	Y [0.33]	110	NTR	nCMS_7	10	MB	12	Y [0.41]	25	GTR
CMS_8	8	MB	22	Y [0.43]	145	GTR	nCMS_8	7	MB	46	Y [0.39]	85	NTR
CMS_9	10	MB	40	Y [0.46]	145	STR	nCMS_9	9	MB	26	Y [0.33]	25	NTR
CMS_10	14	EP II	114	Y [0.44]	130	GTR	nCMS_10	13	EP II	49	Y [0.40]	105	STR

### Characteristics of CMS

Postoperative CMS was first diagnosed on day 1 in 6 patients and on days 1–2 and 2–4 in 2 patients each (Fig. 4). Grading of mutism, ataxia, muscle hypotonia, and irritability is summarized in Fig. 4. In summary, symptoms in all four categories were severe and lasted > 4 weeks in a large proportion of patients (80%, 80%, 60%, and 50%, respectively). First documented major clinical improvement of CMS symptoms occurred at a median of 210 days (range 99–518 days). Mild residual ataxia was present in 80% of children and mild residual speech impairment in 50%.

### Injury pattern and damage burden

Analysis of serial imaging revealed DN injury in all children affected by CMS in this cohort. Unilateral injury of the left DN was observed in 5 cases, whereas the other 5 children had bilateral DN injury. In the non-CMS group, DN injury was observed in 30% of cases (left DN *N* = 1, bilateral DN *N* = 2). The association between uni- or bilateral DN injury and CMS was statistically significant (*P* = 0.0031), but not for bilateral versus unilateral/no DN injury (*P* = 0.0698).

In the CMS group, injury to the SCP was present in 70%. The left SCP was injured in 3 cases, the right SCP in 1 case, and bilateral SCP injury was observed in 3 children. In the non-CMS group, only 2 cases of right SCP injury were found. The association between uni- or bilateral SCP injury and CMS was not statistically significant (*P* = 0.0698).

**Fig. 2:**
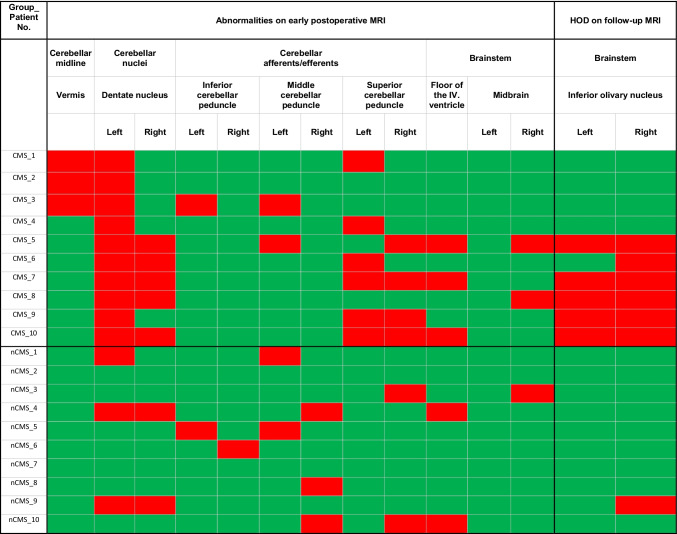
Injury pattern and damage burden of CMS and non-CMS patients

Injury to other posterior fossa structures was observed occasionally in both groups, without differences by tendency or with statistical significance. Injury pattern and damage burden are visualized in Fig. 2.

### Hypertrophic olivary degeneration

**Fig. 3:**
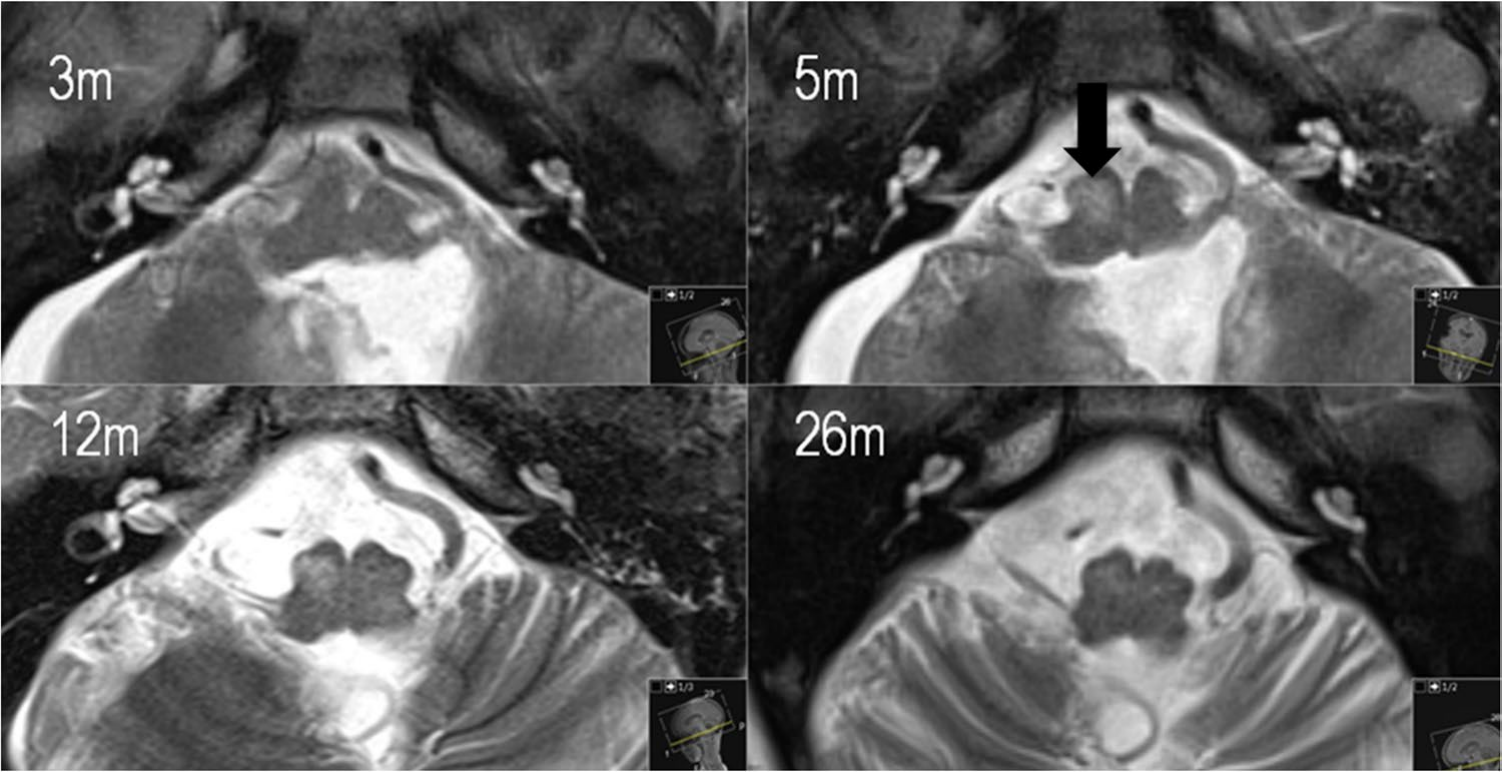
Representative serial postoperative MRI scans of patient CMS_6 (axial T2-weighted sequences, time since tumor resection given in months [m]), with HOD first diagnosed on the MRI scan 5 months after surgery (arrow)

In children with CMS, an abnormality of the inferior olivary nucleus (ION) was detected in 60%, of which 5 cases had bilateral and 1 case had right-sided ION abnormality. ION hypertrophy was first diagnosed in MRI scans obtained after a mean time of 5 months after surgery (range 1 to 8 months) and ION T2 hyperintensity after a mean time of 6.5 months (range 1 to 14 months), respectively. Resolution of ION hypertrophy was first evident on MRI scans performed after a mean time of 13.8 months after surgery (range 7 to 26 months), whereas residual ION T2 hyperintensity persisted throughout radiological follow-up in all cases (Fig. 3). In the non-CMS group, 1 case of right-sided ION abnormality occurred.

**Fig. 4:**
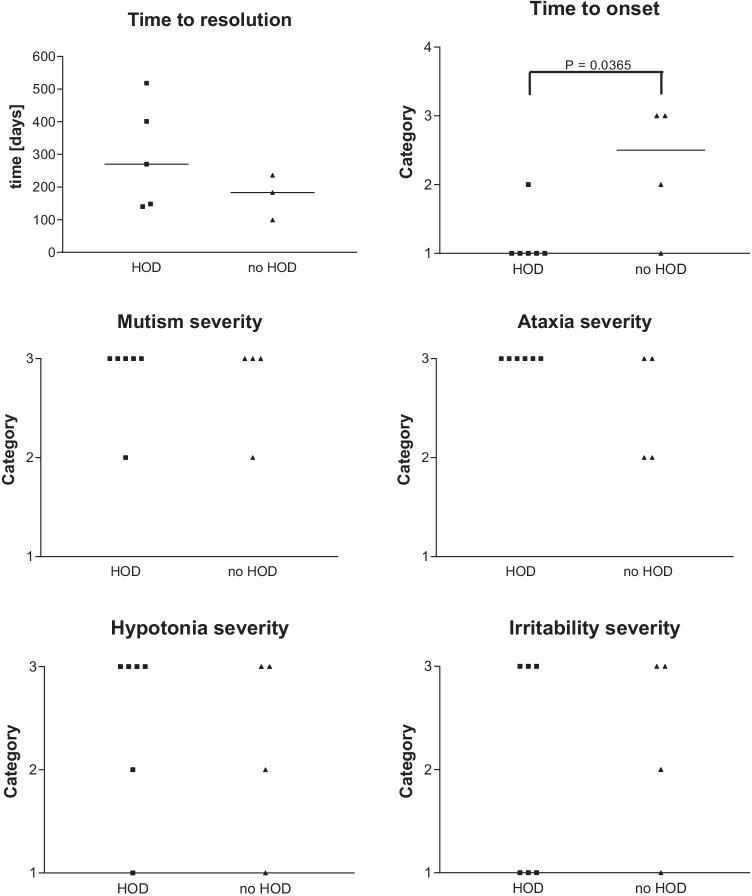
CMS grading according to Robertson et al. [27], with categories for time of onset (category 1 = day 1, 2 = days 1–2, 3 = days 2–4, and 4 =  > day 4) and mutism, ataxia, muscle hypotonia, and irritability (category 1 = mild/ < 1 week, 2 = moderate/1–4 weeks, and 4 = severe/ > 4 weeks)

Presence of CMS significantly correlated with the appearance of uni- or bilateral HOD in this cohort (*P* = 0.0198). The association between bilateral DN injury and uni- or bilateral HON was statistically significant (*P* = 0.0476). CMS occurred earlier in children who later developed HOD (*P* = 0.0365), and by tendency it took longer to resolve (Fig. 4). Grading of CMS symptoms did not show any tendencies when comparing HOD and no-HOD groups. Children tended to be classified into category 3 for all symptoms.

## Discussion

In this study, the postoperative damage burden of anatomical structures involved in the dentato-thalamo-cortical and dentato-rubro-olivary pathways was higher in children who suffered CMS after posterior fossa tumor resection: DN injury significantly correlated with CMS, and SCP injury was associated by tendency. HOD was observed as a dynamic neuroimaging phenomenon in the postoperative course and its presence significantly correlated with CMS and DN injury. Children who later developed HOD had an earlier onset and tended to have longer persistence of CMS. The Rotterdam Risk Score accurately predicted the occurrence of CMS based on preoperative MRI features. The CMS grading according to Robertson et al. offered limited discrimination in this series, as most symptoms were consistently assigned to category 3, i.e., severe (duration > 4 weeks).

**Fig. 5:**
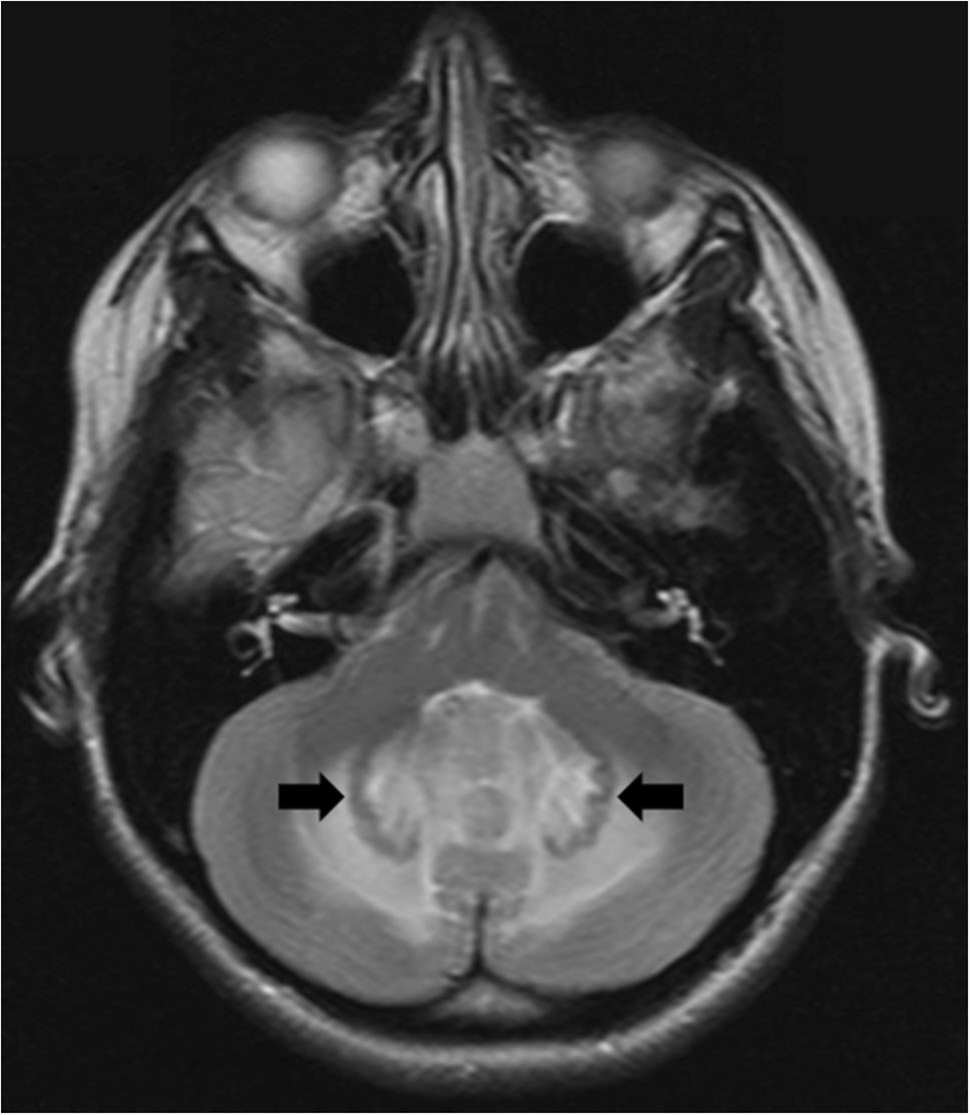
Axial T2-weighted sequence of a preoperative MRI showing the DN on both sides (arrows) within the perifocal edema surrounding a midline cerebellar tumor

Understanding the pathophysiology of CMS is essential to guide neurosurgical strategies to avoid this postoperative morbidity. Previous studies have examined preoperative and postoperative neuroimaging with different but interrelated aims: Preoperative imaging features were used to develop prediction models, one of which was applied in the present study [8, 18, 43]. Analysis of postoperative imaging features indicates the anatomical structures at risk [1, 32, 40]. While damage to the SCP and DN and thus cerebellar efferent injury has been implicated to play a major role in CMS pathophysiology, the present study provides evidence of a “dose dependency”: Especially bilateral DN injury leads to the development of HOD in CMS patients, which is a trans-synaptic pathway degeneration indicating severe neuronal disruption. The majority of reported connections of the DN are projections via the SCP and this is also part of the Guillain-Mollaret triangle [4, 19]. HOD can be interpreted as a surrogate marker of extent or severity of “collateral damage” after posterior fossa surgery. Our additional observation that children with HOD had in retrospect an earlier onset and longer persistence of CMS needs to be confirmed in larger cohorts. However, these findings demand for profound surgical measures to protect the DN and SCP during posterior fossa tumor resections and avoid at least bilateral damage. Figure 5 shows a scenario where the DN on both sides is visible on the preoperative MRI within the perifocal edema surrounding a midline cerebellar tumor — a high-risk constellation for bilateral DN injury.

While preoperative risk prediction can certainly raise the neurosurgeon’s awareness, intraoperative neurophysiological monitoring (IONM) of cerebellar efferent pathways would be an additional major advantage. At present, IONM is well established for supratentorial functional areas, brainstem and cerebello-pontine angle, but not for monitoring of the cerebellum itself. Recently, the first study was published on IONM of the cerebello-dentato-thalamo-cortical pathway [10]. The authors demonstrated that direct stimulation of lobules IV, V, and VI in the anterior cerebellum and lobule VIIB in the posterior cerebellum leads to inhibition of primary motor cortex excitability for transcranial electrical stimulation. In contrast, IONM during resection of supratentorial glioma is a well-established standard technique [30]. Even in IONM-guided glioma surgery, an aggressive resection must be weighed against risk of neurological morbidity, despite the paradigm that degree of resection strongly influences survival in malignant glioma patients. This notion has even greater relevance in children with medulloblastoma, where the prognostic benefit of a GTR over a NTR is smaller than the impact of molecular subgroup affiliation [39]. For WNT, SHH or group 3 medulloblastoma not even GTR versus STR yields a significant benefit. A risk-aversive surgical strategy especially in the region of the DN and SCP thus appears justified in these children. Indeed, we noticed a higher proportion of GTR in the CMS (50%) compared to the non-CMS group (20%), and a significant number of patients in our series were treated before the introduction of routine molecular subgroup analysis. We have since adopted a surgical strategy as outlined by Dhaenens and co-authors [8]. In addition to CMS risk prediction models, preoperative tumor entity or even molecular subgroup prediction models might help to establish a patient-specific strategy, since for example a group 4 medulloblastoma with metastatic disease might benefit from GTR with regard to progression free survival [24, 29, 39]. As a further vision for the future of patient-specific resection strategies, intraoperative real-time analysis of isocitrate dehydrogenase mutation status, an important diagnostic and prognostic marker in glioma, has been established [2]. Once such intraoperative consultations are also possible for mutations relevant in pediatric posterior fossa tumors, the neurosurgeon would have even more information available to find the optimal balance between extent of resection and risk of morbidity, in children most importantly CMS.

Our study has some limitations, in addition to those inherent to retrospective study designs in general. The CMS cohort is relatively small, although within the typical range for single center studies in this field. Our findings help understand CMS pathophysiology and guide surgical strategy, but do not prove causal relationships and do not allow CMS prediction. The impact of avoidance of DN and SCP injury on the incidence of CMS has to be investigated in adequately powered prospective studies, since previous pathophysiological concepts (e.g., the role of the vermis) resulted in equivocal surgical study results [7, 13, 25, 27]. In our opinion, such studies would need to include detailed pre- and postoperative neuroimaging, as the surgical approach itself does not necessarily mean that deep cerebellar structure remains intact: While the telovelar approach “lacks incision of any part of the cerebellum,” retraction or surgical manipulation can of course still cause injury for instance to the DN [37]. Preoperative language or speech impairment, laterality, left/right handedness, swallowing problems, and the spectrum of postoperative speech impairment could not be assessed in our retrospective series with adequate precision and sufficient statistical power. Due to the long period of our study, molecular data is not available for the majority of cases. This is a retrospective study analyzing structural MRI data from routine clinical protocols, and not using advanced neuroimaging research protocols. However, despite its potential advantages, advanced neuroimaging can also introduce further bias and difficulties in comparing results across studies [20]. At least two similar studies conclude that bilateral HOD could serve as an indicator of CMS [3, 23]. However, the authors also encountered limitations with regard to imaging protocols and data on laterality in a retrospective study design [23]. Laterality is an important aspect, as a predilection of damage to the right efferent cerebellar pathways and HOD in the left ION have been implicated in CMS, correlating with left cerebral hemisphere dominance for language in most humans [3]. The incidence of right hemisphere language dominance is 30% in left-handed humans, highlighting the importance of laterality and left/right handedness in future studies on the pathophysiology of CMS [17].

These limitations can only be overcome in our opinion by multicenter prospective approaches, which can provide systematic data and large cohorts. We therefore endeavor to contribute to such studies, such as the Nordic Study of the Cerebellar Mutism Syndrome in Children with Brain Tumours of the Posterior Fossa (NCT02300766), to validate findings and increase the level of evidence [42].

## Conclusion

DN and SCP injury and HOD are consistent findings in this series of children suffering CMS after telovelar approaches to posterior fossa tumors. The Rotterdam Risk Score accurately predicted the occurrence of CMS based on preoperative MRI features. Those who later developed HOD had in retrospect an earlier onset and longer persistence of CMS after tumor resection. ION abnormalities in this cohort followed a time course also reported for other neurological insults to the dentato-rubro-olivary pathway. We propose a “dose dependency” of intraoperative “collateral damage”: Especially bilateral DN injury was associated with HOD in CMS patients, and HOD can thus be interpreted as a surrogate marker of extent or severity of “collateral damage.” These findings demand for profound surgical measures to protect the DN and SCP during posterior fossa tumor resections and avoid at least bilateral damage. IONM of the cerebellar efferent pathways as well as routine use of CMS risk prediction models in combination with preoperative tumor entity or even molecular subgroup prediction models might help to establish a patient-specific strategy with optimal balance between degree of resection and functional integrity.

## Data Availability

The datasets generated during and/or analyzed during the current study are available from the corresponding author on reasonable request.

## References

[1] Ahmadian N, van Baarsen KM, Robe PAJT, Hoving EW (2021). Association between cerebral perfusion and paediatric postoperative cerebellar mutism syndrome after posterior fossa surgery—a systematic review. Child’s Nerv Syst.

[2] Alfaro CM, Pirro V, Keating MF, Hattab EM, Cooks RG, Cohen-Gadol AA (2019). Intraoperative assessment of isocitrate dehydrogenase mutation status in human gliomas using desorption electrospray ionization-mass spectrometry. J Neurosurg.

[3] Avula S, Spiteri M, Kumar R, Lewis E, Harave S, Windridge D, Ong C, Pizer B (2016). Post-operative pediatric cerebellar mutism syndrome and its association with hypertrophic olivary degeneration. Quant Imaging Med Surg.

[4] Beez T, Munoz-Bendix C, Steiger H-J, Hänggi D (2021). Functional tracts of the cerebellum-essentials for the neurosurgeon. Neurosurg Rev.

[5] Boisgontier J, Fillon L, Rutten C, Saitovitch A, Dufour C, Lemaître H, Beccaria K, Blauwblomme T, Levy R, Dangouloff-Ros V, Grévent D, Roux C-J, Grill J, Vinçon-Leite A, Saidoun L, Bourdeaut F, Zilbovicius M, Boddaert N, Puget S (2021) A CBF decrease in the left supplementary motor areas: new insight into postoperative pediatric cerebellar mutism syndrome using arterial spin labeling perfusion MRI. J Cereb Blood Flow Metab 0271678X2110313 10.1177/0271678X211031321 PMC8669281 34259072

[6] Catsman-Berrevoets CE (2017). Cerebellar mutism syndrome: cause and rehabilitation. Curr Opin Neurol.

[7] Dailey AT, McKhann GM, Berger MS (1995). The pathophysiology of oral pharyngeal apraxia and mutism following posterior fossa tumor resection in children. J Neurosurg.

[8] Dhaenens BAE, van Veelen MLC, Catsman-Berrevoets CE (2020) Preoperative prediction of postoperative cerebellar mutism syndrome. Validation of existing MRI models and proposal of the new Rotterdam pCMS prediction model. Child’s Nerv Syst 36(7):1471–1480 10.1007/s00381-020-04535-4 PMC7299925 32072230

[9] Eccles JC, Ito M, Szentágothai J (1967). The cerebellum as a neuronal machine.

[10] Giampiccolo D, Basaldella F, Badari A, Squintani GM, Cattaneo L, Sala F (2021). Feasibility of cerebello-cortical stimulation for intraoperative neurophysiological monitoring of cerebellar mutism. Childs Nerv Syst.

[11] Goyal M, Versnick E, Tuite P, Saint Cyr J, Kucharczyk W, Montanera W, Willinsky R, Mikulis D (2000). Hypertrophic olivary degeneration: metaanalysis of the temporal evolution of MR findings. Am J Neuroradiol.

[12] Goyal M, Versnick E, Tuite P, Cyr JS, Kucharczyk W, Montanera W, Willinsky R, Mikulis D (2000). Hypertrophic olivary degeneration: metaanalysis of the temporal evolution of MR findings. AJNR Am J Neuroradiol.

[13] Grønbæk JK, Wibroe M, Toescu S, Frič R, Thomsen BL, Møller LN, Grillner P, Gustavsson B, Mallucci C, Aquilina K, Fellows GA, Molinari E, Hjort MA, Westerholm-Ormio M, Kiudeliene R, Mudra K, Hauser P, van Baarsen K, Hoving E, Zipfel J, Nysom K, Schmiegelow K, Sehested A, Juhler M, Mathiasen R; CMS study group (2021) Postoperative speech impairment and surgical approach to posterior fossa tumours in children: a prospective European multicentre cohort study. Lancet Child Adolesc Heal 5(11):814–824 10.1016/S2352-4642(21)00274-1 34624241

[14] Gudrunardottir T, Morgan AT, Lux AL, Walker DA, Walsh KS, Wells EM, Wisoff JH, Juhler M, Schmahmann JD, Keating RF, Catsman-Berrevoets CE (2016) Consensus paper on post-operative pediatric cerebellar mutism syndrome: the Iceland Delphi results. 10.1007/s00381-016-3093-3 10.1007/s00381-016-3093-3 27142103

[15] Gudrunardottir T, Sehested A, Juhler M, Grill J, Schmiegelow K (2011). Cerebellar mutism: definitions, classification and grading of symptoms. Child’s Nerv Syst.

[16] Khan RB, Patay Z, Klimo P, Huang J, Kumar R, Boop FA, Raches D, Conklin HM, Sharma R, Simmons A, Sadighi ZS, Onar-Thomas A, Gajjar A, Robinson GW (2021). Clinical features, neurologic recovery, and risk factors of postoperative posterior fossa syndrome and delayed recovery: a prospective study. Neuro Oncol.

[17] Knecht S, Dräger B, Deppe M, Bobe L, Lohmann H, Flöel A, Ringelstein EB, Henningsen H (2000). Handedness and hemispheric language dominance in healthy humans. Brain.

[18] Liu J-F, Dineen RA, Avula S, Chambers T, Dutta M, Jaspan T, MacArthur DC, Howarth S, Soria D, Quinlan P, Harave S, Ong CC, Mallucci CL, Kumar R, Pizer B, Walker DA (2018). Development of a pre-operative scoring system for predicting risk of post-operative paediatric cerebellar mutism syndrome. Br J Neurosurg.

[19] Macht S, Hänggi D, Turowski B (2009). Hypertrophic olivary degeneration following pontine cavernoma hemorrhage: a typical change accompanying lesions in the Guillain-Mollaret triangle. Klin Neuroradiol.

[20] O’Donnell LJ, Pasternak O (2015). Does diffusion MRI tell us anything about the white matter? An overview of methods and pitfalls. Schizophr Res.

[21] Onen MR, Moore K, Cikla U, Ucer M, Schmidt B, Field AS, Baskaya MK (2018). Hypertrophic olivary degeneration: neurosurgical perspective and literature review. World Neurosurg.

[22] Pajtler KW, Mack SC, Ramaswamy V, Smith CA, Witt H, Smith A, Hansford JR, von Hoff K, Wright KD, Hwang E, Frappaz D, Kanemura Y, Massimino M, Faure-Conter C, Modena P, Tabori U, Warren KE, Holland EC, Ichimura K, Giangaspero F, Castel D, von Deimling A, Kool M, Dirks PB, Grundy RG, Foreman NK, Gajjar A, Korshunov A, Finlay J, Gilbertson RJ, Ellison DW, Aldape KD, Merchant TE, Bouffet E, Pfister SM, Taylor MD (2017). The current consensus on the clinical management of intracranial ependymoma and its distinct molecular variants. Acta Neuropathol.

[23] Patay Z, Enterkin J, Harreld JH, Yuan Y, Löbel U, Rumboldt Z, Khan R, Boop F (2014). MR imaging evaluation of inferior olivary nuclei: comparison of postoperative subjects with and without posterior fossa syndrome. AJNR Am J Neuroradiol.

[24] Perreault S, Ramaswamy V, Achrol AS, Chao K, Liu TT, Shih D, Remke M, Schubert S, Bouffet E, Fisher PG, Partap S, Vogel H, Taylor MD, Cho YJ, Yeom KW (2014). MRI surrogates for molecular subgroups of medulloblastoma. Am J Neuroradiol.

[25] Pettersson SD, Kitlinski M, Miękisiak G, Ali S, Krakowiak M, Szmuda T (2021) Risk factors for postoperative cerebellar mutism syndrome in pediatric patients: a systematic review and meta-analysis. J Neurosurg Pediatr 1–9 10.3171/2021.11.PEDS21445 34972081

[26] Reed-Berendt R, Phillips B, Picton S, Chumas P, Warren D, Livingston JH, Hughes E, Morrall MCHJ (2014). Cause and outcome of cerebellar mutism: evidence from a systematic review. Child’s Nerv Syst.

[27] Robertson PL, Muraszko KM, Holmes EJ, Sposto R, Packer RJ, Gajjar A, Dias MS, Allen JC (2006) Incidence and severity of postoperative cerebellar mutism syndrome in children with medulloblastoma: a prospective study by the Children’s Oncology Group. J Neurosurg 105 Pediat(Suppl 6):444–451 10.3171/ped.2006.105.6.444 17184075

[28] Di Rocco C, Chieffo D, Frassanito P, Caldarelli M, Massimi L, Tamburrini G (2011). Heralding cerebellar mutism: evidence for pre-surgical language impairment as primary risk factor in posterior fossa surgery. The Cerebellum.

[29] Rumboldt Z, Camacho DLA, Lake D, Welsh CT, Castillo M (2006). Apparent diffusion coefficients for differentiation of cerebellar tumors in children. Am J Neuroradiol.

[30] Sanai N, Berger MS (2010) Intraoperative stimulation techniques for functional pathway preservation and glioma resection. Neurosurg Focus 28(2):E1 10.3171/2009.12.FOCUS09266 20121436

[31] Schmahmann JD (2019) Pediatric post-operative cerebellar mutism syndrome, cerebellar cognitive affective syndrome, and posterior fossa syndrome: historical review and proposed resolution to guide future study. Child’s Nerv Syst l 10.1007/s00381-019-04253-6 PMC7020253 31240391

[32] Soelva V, Hernáiz Driever P, Abbushi A, Rueckriegel S, Bruhn H, Eisner W, Thomale U-W (2013). Fronto-cerebellar fiber tractography in pediatric patients following posterior fossa tumor surgery. Child’s Nerv Syst.

[33] Sotelo C (2008). Viewing the cerebellum through the eyes of Ramón Y Cajal. The Cerebellum.

[34] Stoodley CJ, MacMore JP, Makris N, Sherman JC, Schmahmann JD (2016) Location of lesion determines motor vs. cognitive consequences in patients with cerebellar stroke. NeuroImage Clin 12:765–775 10.1016/j.nicl.2016.10.013 PMC5079414 27812503

[35] Stoodley CJ, Schmahmann JD (2009). Functional topography in the human cerebellum: a meta-analysis of neuroimaging studies. Neuroimage.

[36] Stoodley CJ, Valera EM, Schmahmann JD (2010). An fMRI study of intra-individual functional topography in the human cerebellum. Behav Neurol.

[37] Tanriover N, Ulm AJ, Rhoton AL, Yasuda A (2004). Comparison of the transvermian and telovelar approaches to the fourth ventricle. J Neurosurg.

[38] Taylor MD, Northcott PA, Korshunov A, Remke M, Cho Y-J, Clifford SC, Eberhart CG, Parsons DW, Rutkowski S, Gajjar A, Ellison DW, Lichter P, Gilbertson RJ, Pomeroy SL, Kool M, Pfister SM (2012) Molecular subgroups of medulloblastoma: the current consensus. Acta Neuropathol 123(4):465–472 10.1007/s00401-011-0922-z PMC3306779 22134537

[39] Thompson EM, Hielscher T, Bouffet E, Remke M, …, Taylor MD (2016) Prognostic value of medulloblastoma extent of resection after accounting for molecular subgroup: a retrospective integrated clinical and molecular analysis. Lancet Oncol 17(4):484–495 10.1016/S1470-2045(15)00581-1 PMC4907853 26976201

[40] Toescu SM, Hales PW, Aquilina K, Clark CA (2018). Quantitative MRI in post-operative paediatric cerebellar mutism syndrome. Eur J Radiol.

[41] Wang H, Wang Y, Wang R, Li Y, Wang P, Li J, Du J (2019). Hypertrophic olivary degeneration: a comprehensive review focusing on etiology. Brain Res.

[42] Wibroe M, Cappelen J, Castor C, Clausen N, Grillner P, Gudrunardottir T, Gupta R, Gustavsson B, Heyman M, Holm S, Karppinen A, Klausen C, Lönnqvist T, Mathiasen R, Nilsson P, Nysom K, Persson K, Rask O, Schmiegelow K, Sehested A, Thomassen H, Tonning-Olsson I, Zetterqvist B, Juhler M (2017) Cerebellar mutism syndrome in children with brain tumours of the posterior fossa. BMC Cancer 17(1):439 10.1186/s12885-017-3416-0 PMC5480181 28637445

[43] Zhang H, Liao Z, Hao X, Han Z, Li C, Gong J, Liu W, Tian Y (2019). Establishing reproducible predictors of cerebellar mutism syndrome based on pre-operative imaging. Childs Nerv Syst.

